# Balanced Standing on One Foot of Biped Robot Based on Three-Particle Model Predictive Control

**DOI:** 10.3390/biomimetics7040244

**Published:** 2022-12-16

**Authors:** Yong Yang, Jiyuan Shi, Songrui Huang, Yuhong Ge, Wenhan Cai, Qingkai Li, Xueying Chen, Xiu Li, Mingguo Zhao

**Affiliations:** 1 Shenzhen International Graduate School, Tsinghua University, Shenzhen 518055, China; 2 Department of Automation, Tsinghua University, Beijing 100084, China

**Keywords:** model predictive control, whole-body control, biped robot balance

## Abstract

Balancing is a fundamental task in the motion control of bipedal robots. Compared to two-foot balancing, one-foot balancing introduces new challenges, such as a smaller supporting polygon and control difficulty coming from the kinematic coupling between the center of mass (CoM) and the swinging leg. Although nonlinear model predictive control (NMPC) may solve this problem, it is not feasible to implement it on the actual robot because of its large amount of calculation. This paper proposes the three-particle model predictive control (TP-MPC) approach. It combines with the hierarchical whole-body control (WBC) to solve the one-leg balancing problem in real time. The bipedal robot’s torso and two legs are modeled as three separate particles without inertia. The TP-MPC generates feasible swing leg trajectories, followed by the WBC to adjust the robot’s center of mass. Since the three-particle model is linear, the TP-MPC requires less computational cost, which implies real-time execution on an actual robot. The proposed method is verified in simulation. Simulation results show that our method can resist much larger external disturbance than the WBC-only control scheme.

## 1. Introduction

### 1.1. Background

Motion control of a bipedal robot is a typical multi-task control problem. The robot has to keep its balance while moving or standing, interact with the environment, and complete tasks assigned by the user. Among the various tasks, balancing under external disturbance is a fundamental one for the robot to complete other tasks. Therefore, the balance control of a bipedal robot while walking or standing on both feet has been widely studied in recent years. However, the situation where robots stand on one leg and keep their balance has received little attention. Examples of such a situation include a humanoid trying to reach a distant object with its arms or a bipedal robot kicking a football. Compared with balancing on both feet, one-foot balancing presents new challenges, such as significantly reduced support polygons and reduced overall system stability. Furthermore, if the swing leg is heavy enough, its motion may significantly affect the motion of the entire robot [1]. In this sense, the problem of robot balance control with one foot is similar to that of robot balance control with two arms [2]. From a physical point of view, maintaining the balance of a biped robot with its arms reduces the difficulty of the control problem because the robot’s supporting polygon remains unchanged, while extra degrees of freedom can be used to accomplish the balance task [3]. Therefore, the single-leg balancing stance method can be applied to dual-arm robots and vice versa.

### 1.2. Motivation

A bipedal robot’s balancing on one foot is a typical multi-task control problem. The robot must keep the torso and the stance foot stable, move the swing foot properly, and conform to external and internal constraints. The whole-body control (WBC) method has been widely adopted in the balancing control of bipedal robots due to its capabilities to generate dynamically feasible control outputs and to handle various tasks and constraints [4]. However, the WBC only considers the current states of the robot and cannot handle strongly infeasible reference trajectories [5]. The model predictive control (MPC) is commonly used in conjunction with WBC to complement these insufficiencies [6]. In case of large external disturbance, the MPC foresees the robot’s future states according to a specific dynamical model and acts as an online re-planner for the WBC. However, the full dynamics of a one-foot standing robot are high degrees of freedom and strongly nonlinear. Therefore, an MPC employing full dynamics has to solve a complicated nonlinear optimization problem. Thus, the full-dynamics MPC is computationally expensive and cannot be executed in real time on an actual robot [7,8,9,10,11].

Current MPC algorithms for legged robots usually adopt simplified models [12], for example, the linear inverted pendulum (LIP) model [2,13,14], the single particle model [15] or the single rigid body (SRB) model [6], to balance between model precision and computation time. In the case of balancing on one foot, these models are unsuitable because they adopt the dynamics model of a particle or a rigid body. Thus, the motion of the swinging leg cannot be predicted in MPC. In this research, we propose to adopt a three-particle model in the model predictive control, which is called the three-particle model predictive control (TP-MPC) approach. It considers the motion of the swing leg and requires far less computation time than a full-dynamics MPC. As a result, the TP-MPC combined with the WBC will solve the problem of one-foot balance control for practical biped robots with real-time computing requirements.

### 1.3. Related Work

Simplified models have been widely used in the balance control of bipedal robots. For example, Vukobratovic et al. first proposed the zero moment point (ZMP) concept and applied it to balance a bipedal robot [16]. Next, Kajita et al. proposed the LIP model, which simplifies the biped robot into a single mass point moving horizontally and connected to the ground by a massless retractable stick [17]. Finally, Pratt et al. extended the LIP model to a Linear Flywheel Inverted Pendulum Model, which takes the robot’s angular momentum into account [18]. Their method can resist a large external disturbance by computing the robot’s Capture Point and Capture Region. Li et al. used the momentum to control the balance of a biped robot, and they found that linear momentum was more critical than angular momentum in the balance control task. When the two momentum tasks cannot be satisfied simultaneously due to a significant disturbance, the linear momentum will be given priority [19].

WBC has also been applied to the bipedal robot’s balancing. Xie et al. used the hierarchical WBC to maintain a balance of a standing biped robot [20]. Their algorithm can execute on an onboard computer in real time by optimizing the computing process. However, their control scheme needs a planner to generate feasible trajectories followed by the WBC to resist large external disturbances. Kim et al. proposed a new whole-body control approach called whole-body motion control [21]. It can realize dynamic walking on a bipedal robot with a passive ankle and maintain balance under external disturbance.

Model predictive control repeatedly solves a finite-horizon optimal control problem starting from the system’s current states [22]. Moreover, the MPC can prepare for future motions in advance. Therefore, it is very suitable for highly dynamic motion control of legged robots. Li et al. applied a single rigid body model predictive control to a biped robot with light legs [23]. The MPC generates optimal contact forces and torques at each foot, which are converted to joint torque commands through contact Jacobians. Their method enables the robot to walk at a speed of 1.6 m/s on complex terrains and achieve a wide range of dynamic motions. Luo et al. proposed a three-mass model predictive control method for gait control of biped robots [24]. Their planar three-mass model considers the robot’s ZMP and angular momentum. The swing leg trajectories are generated by heuristics rather than the MPC. Therefore, their method did not utilize the swing leg to balance the robot.

From a biomimicry point of view, swinging the legs is crucial for the balanced control of the torso [1]. Boston Dynamics released a video in 2013 of using swinging legs and arms to adjust posture but did not release relevant technical details [25]. To the best of our knowledge, other than the two works mentioned above, no literature has been published on the use of swinging legs to control the balance of biped robots.To the best of our knowledge, literature has yet to be published on the balance control of biped robots using the swinging leg.

### 1.4. Contribution

The main contributions of this paper are as follows:

(1) The proposed TP-MPC method can generate feasible swing leg trajectories that balance the robot while standing on one foot. The WBC tracks the generated swing leg trajectories. As a result, the overall control scheme can resist large external disturbances.
(2) The TP-MPC catches the main effects of the swing leg motion while being simple enough to operate at the same frequency as the WBC.

The overall structure of this paper is as follows: In Section 2, the three-particle model and the TP-MPC are derived. Section 3 introduces the hierarchical whole-body control approach and the overall control scheme. Section 4 discusses the simulation setups and results. Finally, Section 5 is for the conclusions and future work.

## 2. Three-Particle Model Predictive Control

In this section, we derive the three-particle model predictive control method. First, we introduce the simplified three-particle model for a bipedal robot standing on a single foot. Our MPC method requires to solve repeatedly a discrete, finite-horizon optimal control problem as follows:

(1) $$\min_{X,U}=\sum_{k=0}^{n-1}{∥\begin{array}{c} x_{k+1}-x_{k+1,ref} \end{array}∥}_{Q_{k}}+{∥\begin{array}{c} u_{k} \end{array}∥}_{R_{k}}$$

(2) $$\begin{array}{c} s.t.\;x_{k+1}=A_{k}x_{k}+B_{k}u_{k},\;k=0,1,\cdots ,n-1 \end{array}$$

(3) $$\begin{array}{c} {\mathrm{lb}}_{k}\leq C_{k}u_{k}\leq {\mathrm{ub}}_{k},\;k=0,1,\cdots ,n-1 \end{array}$$

where *n* is the MPC’s prediction length; $x_{k}$ and $x_{k,ref}$ are the actual state and reference state at step *k*, $u_{k}$ is the input of step *k*, and $Q_{k}$ and $R_{k}$ are the weight matrices, ${∥\begin{array}{c} \lambda \end{array}∥}_{\Gamma }=\lambda^{⊤}\Gamma \lambda$ means the weighted value of λ of weight Γ. Equations (2) and (3) are equality and inequality constraints, respectively.

### 2.1. Three-Particle Simplified Model

**Figure 1:**
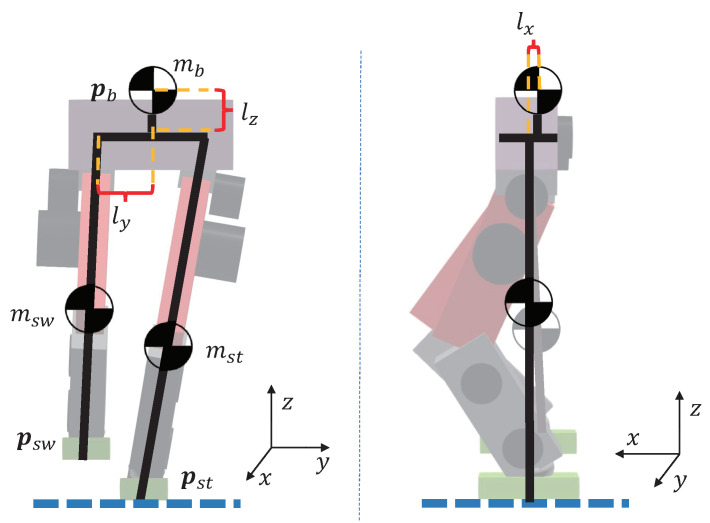
Three-Particle Model.

Figure 1 shows the three-particle simplified model. The stance leg of the biped robot is fixed, and the position of the robot’s center of mass can be adjusted by moving the torso and the swing leg to fall near the desired position. Here, we simplified the legs and the torso into three mass points without rotational inertia. In most cases, the torso’s center of mass is located directly above the midpoint of the two hip joints but has an offset in the sagittal direction.

As shown in Figure 1, $p_{st}={\left[\begin{array}{ccc} p_{st,x} & p_{st,y} & p_{st,z} \end{array}\right]}^{⊤}$, $p_{sw}={\left[\begin{array}{ccc} p_{sw,x} & p_{sw,y} & p_{sw,z} \end{array}\right]}^{⊤}$ are the positions of the end of the stance leg and the swing leg in the world coordinate system, $p_{b}={\left[\begin{array}{ccc} p_{b,x} & p_{b,y} & p_{b,z} \end{array}\right]}^{⊤}$ is the position of the torso in the world coordinate system, ${\dot{p}}_{st}={\left[\begin{array}{ccc} {\dot{p}}_{st,x} & {\dot{p}}_{st,y} & {\dot{p}}_{st,z} \end{array}\right]}^{⊤}$, ${\dot{p}}_{sw}={\left[\begin{array}{ccc} {\dot{p}}_{sw,x} & {\dot{p}}_{sw,y} & {\dot{p}}_{sw,z} \end{array}\right]}^{⊤}$ are the velocities of the end of the stance leg and the swing leg in the world coordinate system, ${\dot{p}}_{b}={\left[\begin{array}{ccc} {\dot{p}}_{b,x} & {\dot{p}}_{b,y} & {\dot{p}}_{b,z} \end{array}\right]}^{⊤}$ is the velocity of the torso in the world coordinate system. $m_{st}$, $m_{sw}$, and $m_{b}$ denote the mass of the stance leg, swing leg, and torso, respectively. In general, bipedal robots are symmetrical in the *y* direction, so the hip joint’s distance from the hip’s center in the *y* direction is $l_{y}$. Let the distance between the torso center of mass and the hip plane be $l_{z}$, and $l_{x}$ is the distance between the torso center of mass in the *x* direction and the hip motor of both legs.

Let $p_{m,st}$,$p_{m,sw}$ be the center of mass position of the stance leg and the swing leg, respectively. To simplify the model, we assume that each leg’s center of mass lies on the midpoint of the hip and foot, which means

(4) $$p_{m,st}=\frac{1}{2}\left[\begin{array}{c} p_{b,x}+l_{x}+p_{st,x}\\ p_{b,y}+l_{y}+p_{st,y}\\ p_{b,z}-l_{z}+p_{st,z} \end{array}\right]$$

(5) $$p_{m,sw}=\frac{1}{2}\left[\begin{array}{c} p_{b,x}+l_{x}+p_{sw,x}\\ p_{b,y}-l_{y}+p_{sw,y}\\ p_{b,z}-l_{z}+p_{sw,z} \end{array}\right]$$

The robot system’s center of mass can be derived as follows:

(6) $$mp_{CoM}=m_{b}p_{b}+m_{st}p_{m,st}+m_{sw}p_{m,sw}$$

where $m=m_{b}+m_{st}+m_{sw}$ represents the robot’s total mass. Therefore, the robot’s center of mass at time *k* is:

(7) $$p_{CoM,k}=\frac{m_{b}p_{b,k}+m_{st}p_{m,st,k}+m_{sw}p_{m,sw,k}}{m}$$

Let $x={\left[\begin{array}{cccc} p_{b}^{⊤} & p_{sw}^{⊤} & {\dot{p}}_{b}^{⊤} & {\dot{p}}_{sw}^{⊤} \end{array}\right]}^{⊤}$ be the state variables of the system, $u={\left[\begin{array}{cc} {\ddot{p}}_{b}^{⊤} & {\ddot{p}}_{sw}^{⊤} \end{array}\right]}^{⊤}$ be the control input. For the model predictive control design, using Taylor expansion, we obtain the state equation of the discrete-time linear system as:

(8) xk+1=I3×303×3ΔtI3×303×303×3I3×303×3ΔtI3×303×303×3I3×303×303×303×303×3I3×3︸Axk+12Δt2I6×6ΔtI6×6I3×303×3ΔtI3×303×303×3I3×303×3ΔtI3×303×303×3I3×303×303×303×303×3I3×3︸Buk

Substituting Equations (4) and (5) into Equation (7) yields the location of the biped robot’s center of mass:

(9) pCoM,k=1mm1I3×3m2I3×303×6mstxst+(msw+mst)lxmstyst+(msw−mst)lymstzst−(msw+mst)lz︸Cxk+12mmstxst+(msw+mst)lxmstyst+(msw−mst)lymstzst−(msw+mst)lz︸D

where $m_{1}=\frac{1}{2}\left(m_{sw}+m_{st}\right)+m_{b}$ and $m_{2}=\frac{1}{2}m_{sw}$.

### 2.2. Tasks

For the one-foot balance control of a biped robot, the primary task is to minimize the position and velocity tracking errors of the robot’s center of mass. The position tracking error of the swing leg should also be minimized. Moreover, the system input should be regularized to avoid excessive joint torques. Therefore, we formulate the cost function of the MPC as follows:

(10) $$\begin{array}{cc} J=\min_{U}\sum_{k=1}^{n}\; & {∥\begin{array}{c} p_{CoM,k}-p_{CoM,ref,k} \end{array}∥}_{Q}+{∥\begin{array}{c} {\dot{p}}_{CoM,k} \end{array}∥}_{T}+{∥\begin{array}{c} {\bar{p}}_{sw,k}-{\bar{p}}_{sw,ref,k} \end{array}∥}_{S}+\\ \; & {∥\begin{array}{c} {\bar{p}}_{b,k}-{\bar{p}}_{b,ref,k} \end{array}∥}_{W}+{∥\begin{array}{c} u_{k-1} \end{array}∥}_{R} \end{array}$$

where ${∥\begin{array}{c} p_{CoM,k}-p_{CoM,ref,k} \end{array}∥}_{Q}$ is the CoM position tracking task where $p_{ref,k}$ is the reference trajectory of the CoM; ${∥\begin{array}{c} {\dot{p}}_{CoM,k} \end{array}∥}_{T}$ is the CoM velocity tracking task, where the desired velocity is set to 0; ${∥\begin{array}{c} {\bar{p}}_{sw,k}-{\bar{p}}_{sw,ref,k} \end{array}∥}_{S}$ is the position and velocity tracking task of the swing leg, where ${\bar{p}}_{sw,k}$ and ${\bar{p}}_{sw,ref,k}$ are the actual and desired trajectory of the swing leg, respectively; ${∥\begin{array}{c} {\bar{p}}_{b,k}-{\bar{p}}_{b,ref,k} \end{array}∥}_{W}$ is the position and velocity tracking task of the torso; ${\bar{p}}_{b,k}$ and ${\bar{p}}_{b,ref,k}$ are the actual trajectory and the desired trajectory of the torso; ${∥\begin{array}{c} u_{k} \end{array}∥}_{R}$ is the input penalty term; $Q\in R^{3\times 3}$, $T\in R^{3\times 3}$, $S\in R^{6\times 6}$, $W\in R^{6\times 6}$, $R\in R^{6\times 6}$ are the correspondent weight matrices.

Substituting Equation (8) into Equation (9), the position of the robot’s center of mass can be expressed as:

(11) $$p_{CoM,k}=Cx_{k}+D=CAx_{k-1}+CBu_{k-1}+D$$

The velocity of the center of mass can be obtained by taking the derivative of Equation (6). Then, its discrete form can be obtained:

(12) $${\dot{p}}_{CoM,k}=Fx_{k}=FAx_{k-1}+FBu_{k-1}$$

where

(13) $$F=\left[\begin{array}{cccc} 0_{3\times 3} & 0_{3\times 3} & \frac{m_{1}}{m}I_{3\times 3} & \frac{m_{2}}{m}I_{3\times 3} \end{array}\right]$$

The position and velocity of the swing leg can be derived by ${\bar{p}}_{sw,k}=Ex_{k}$, where

(14) $$E=\left[\begin{array}{cccc} 0_{3\times 3} & I_{3\times 3} & 0_{3\times 3} & 0_{3\times 3}\\ 0_{3\times 3} & 0_{3\times 3} & 0_{3\times 3} & I_{3\times 3} \end{array}\right]$$

is the selection matrix. Similarly, for the torso position and velocity, ${\bar{p}}_{b,k}=Gx_{k}$, where

(15) $$G=\left[\begin{array}{cccc} I_{3\times 3} & 0_{3\times 3} & 0_{3\times 3} & 0_{3\times 3}\\ 0_{3\times 3} & 0_{3\times 3} & I_{3\times 3} & 0_{3\times 3} \end{array}\right]$$

### 2.3. Constraints

In order to ensure the real-time performance of the model predictive control algorithm, there should not be too many constraints in the MPC. Here, we only constrain the length of the swing leg to avoid the kinematic singularity. Since the Cartesian distance $L=\sqrt{{\left(x-x_{1}\right)}^{2}+{\left(y-y_{1}\right)}^{2}+{\left(z-z_{1}\right)}^{2}}$ is a nonlinear function of coordinates x,y,z, we use the Manhattan distance instead. The swing leg’s length constraint is written as

(16) $$\left|\begin{array}{c} x_{b}-x_{sw} \end{array}\right|+\left|\begin{array}{c} y_{b}-y_{sw} \end{array}\right|+\left|\begin{array}{c} z_{b}-z_{sw} \end{array}\right|\leq L_{leg}$$

Take the intermediate variable $s_{leg}={\left[\begin{array}{ccc} s_{x} & s_{y} & s_{z} \end{array}\right]}^{⊤}$, which satisfies

(17) $$\left\{\begin{array}{c} s_{leg}\geq p_{b}-p_{sw}\\ s_{leg}\geq p_{sw}-p_{b} \end{array}\right.$$

Then, $\left|\begin{array}{c} s_{leg} \end{array}\right|\leq L_{leg}$ is equivalent to the constraints of Equation (16). The matrix form of the constraints can be derived as:

(18) $$\left\{\begin{array}{c} s_{leg}\geq C_{1}x_{k}\\ s_{leg}\geq C_{2}x_{k}\\ 1^{⊤}s_{leg}\leq L_{leg} \end{array}\right.$$

where

(19) $$C_{1}=\left[\begin{array}{cccc} I_{3\times 3} & -I_{3\times 3} & 0_{3\times 3} & 0_{3\times 3} \end{array}\right]$$

(20) $$C_{2}=\left[\begin{array}{cccc} -I_{3\times 3} & I_{3\times 3} & 0_{3\times 3} & 0_{3\times 3} \end{array}\right]$$

(21) $$1={\left[\begin{array}{ccc} 1 & 1 & 1 \end{array}\right]}^{⊤}$$

### 2.4. MPC Optimization Problem

#### 2.4.1. Tasks

In order to improve the calculation efficiency, we eliminate the intermediate variables by substituting system dynamics into the cost function. Equation (8) can be expanded to

(22) $$\begin{array}{cc} x_{1}= & Ax_{0}+Bu_{0}\\ x_{2}= & Ax_{1}+Bu_{1}=A^{2}x_{0}+ABu_{0}+Bu_{1}\\ \; & \vdots \\ x_{n}= & Ax_{n-1}+Bu_{n-1}=A^{n}x_{0}+A^{n-1}Bu_{0}+\cdots +ABu_{n-2}+Bu_{n-1} \end{array}$$

Rewrite Equation (22) into matrix form as

(23) $$\underset{X}{\underset{︸}{\left[\begin{array}{c} x_{1}\\ x_{2}\\ \vdots \\ x_{n} \end{array}\right]}}=\underset{\tilde{A}}{\underset{︸}{\left[\begin{array}{c} A\\ A^{2}\\ \vdots \\ A^{n} \end{array}\right]}}x_{0}+\underset{\tilde{B}}{\underset{︸}{\left[\begin{array}{cccc} B & 0 & \cdots & 0\\ AB & B & \cdots & 0\\ \vdots & \vdots & \ddots & \vdots \\ A^{n-1}B & A^{n-2}B & \cdots & B \end{array}\right]}}\underset{U}{\underset{︸}{\left[\begin{array}{c} u_{0}\\ u_{1}\\ \vdots \\ u_{n-1} \end{array}\right]}}$$

Then, by replacing the matrices with $X,\tilde{A},\tilde{B}$ and U, Equation (23) can be rewritten as:

(24) $$X=\tilde{A}x_{0}+\tilde{B}U$$

Similarly, for the robot’s center of mass position,

(25) $$P_{CoM}=\tilde{C}X+\tilde{D}$$

where $\tilde{C}=\mathrm{diag}\left(C,C,\cdots ,C\right)\in R^{3n\times 12n}$, $\tilde{D}={\left[\begin{array}{c} D^{⊤},D^{⊤},\cdots ,D^{⊤} \end{array}\right]}^{⊤}\in R^{3n\times 1}$. The CoM velocity can be written as:

(26) $${\dot{P}}_{CoM}=\tilde{F}X$$

where

(27) $$\tilde{F}=\mathrm{diag}\left(F,F,\cdots ,F\right)\in R^{3n\times 12n}$$

The position and velocity equations of the swing leg can be written as:

(28) $${\bar{P}}_{sw}=\tilde{E}X$$

where

(29) $$\tilde{E}=\mathrm{diag}\left(E,E,\cdots ,E\right)\in R^{6n\times 12n}$$

Similarly, the position and velocity equations of the torso can be written as:

(30) $${\bar{P}}_{b}=\tilde{G}X$$

where

(31) $$\tilde{G}=\mathrm{diag}\left(G,G,\cdots ,G\right)\in R^{6n\times 12n}$$

The stacked acceleration vector is as follows:

(32) $$U={\left[\begin{array}{c} u_{0}^{⊤},u_{1}^{⊤},\cdots ,u_{n-1}^{⊤} \end{array}\right]}^{⊤}\in R^{6n\times 1}$$

Therefore, the cost function of Equation (10) can be rewritten as

(33) $$\begin{array}{cc} \tilde{J}=\min_{U}\; & {∥\begin{array}{c} P_{CoM}-P_{CoM,ref} \end{array}∥}_{\tilde{Q}}+{∥\begin{array}{c} {\dot{P}}_{CoM} \end{array}∥}_{\tilde{T}}+\\ \; & {∥\begin{array}{c} {\bar{P}}_{sw}-{\bar{P}}_{sw,ref} \end{array}∥}_{\tilde{S}}+{∥\begin{array}{c} {\bar{P}}_{b}-{\bar{P}}_{b,ref} \end{array}∥}_{\tilde{W}}+{∥\begin{array}{c} U \end{array}∥}_{\tilde{R}} \end{array}$$

where

(34) $$\tilde{Q}=\mathrm{diag}\left(Q,Q\cdots Q\right)\in R^{3n\times 3n}$$

(35) $$\tilde{T}=\mathrm{diag}\left(T,T\cdots T\right)\in R^{3n\times 3n}$$

(36) $$\tilde{S}=\mathrm{diag}\left(S,S\cdots S\right)\in R^{6n\times 6n}$$

(37) $$\tilde{W}=\mathrm{diag}\left(W,W\cdots W\right)\in R^{6n\times 6n}$$

(38) $$\tilde{R}=\mathrm{diag}\left(R,R\cdots R\right)\in R^{6n\times 6n}$$

By eliminating the intermediate variables, Equation (33) can be rewritten as

(39) $$\begin{array}{cc} \tilde{J}=\min_{U}\; & {∥\begin{array}{c} \tilde{C}\tilde{A}x_{0}+\tilde{C}\tilde{B}U+\tilde{D}-P_{CoM,ref} \end{array}∥}_{\tilde{Q}}+{∥\begin{array}{c} \tilde{F}\tilde{A}x_{0}+\tilde{F}\tilde{B}U \end{array}∥}_{\tilde{T}}+\\ \; & {∥\begin{array}{c} \tilde{E}\tilde{A}x_{0}+\tilde{E}\tilde{B}U-{\bar{P}}_{sw,ref} \end{array}∥}_{\tilde{S}}+{∥\begin{array}{c} \tilde{G}\tilde{A}x_{0}+\tilde{G}\tilde{B}U-{\bar{P}}_{b,ref} \end{array}∥}_{\tilde{W}}+{∥\begin{array}{c} U \end{array}∥}_{\tilde{R}} \end{array}$$

#### 2.4.2. Constraints

According to Equation (3), the stacked form of the swing leg length constraint can be derived as

(40) $$\left\{\begin{array}{c} {\tilde{1}}^{⊤}{\tilde{C}}_{1}\tilde{B}U\leq {\tilde{L}}_{leg}-{\tilde{1}}^{⊤}{\tilde{C}}_{1}\tilde{A}x_{0}\\ {\tilde{1}}^{⊤}{\tilde{C}}_{2}\tilde{B}U\leq {\tilde{L}}_{leg}-{\tilde{1}}^{⊤}{\tilde{C}}_{2}\tilde{A}x_{0} \end{array}\right.$$

where

(41) $${\tilde{C}}_{1}=\mathrm{diag}\left(C_{1},C_{1},\cdots ,C_{1}\right)\in R^{3n\times 12n}$$

(42) $${\tilde{C}}_{2}=\mathrm{diag}\left(C_{2},C_{2},\cdots ,C_{2}\right)\in R^{3n\times 12n}$$

(43) $${\tilde{L}}_{leg}={\left[\begin{array}{c} L_{leg},L_{leg},\cdots ,L_{leg} \end{array}\right]}^{⊤}\in R^{n\times 1}$$

(44) $$\tilde{1}=\mathrm{diag}\left(1,1,\cdots ,1\right)\in R^{3n\times n}$$

#### 2.4.3. The Quadratic Programming Problem

The MPC problem can finally be transformed into the following quadratic optimization problem, which can be solved by off-the-shell QP solvers:

(45) $$\begin{array}{cc} \min_{U} & \;\frac{1}{2}U^{⊤}H_{qp}U+g_{qp}^{⊤}U\\ s.t.\; & \;{\mathrm{lb}}_{qp}\leq A_{qp}U\leq {\mathrm{ub}}_{qp} \end{array}$$

where

(46) $$H_{qp}=H_{1}+H_{2}+H_{3}+H_{4}+H_{5}$$

(47) $$g_{qp}=g_{1}+g_{2}+g_{3}+g_{4}+g_{5}$$

(48) $$A_{qp}=\left[\begin{array}{c} {\tilde{1}}^{⊤}{\tilde{C}}_{1}\tilde{B}U\\ {\tilde{1}}^{⊤}{\tilde{C}}_{2}\tilde{B}U \end{array}\right]$$

(49) $${\mathrm{ub}}_{qp}=\left[\begin{array}{c} {\tilde{L}}_{leg}-{\tilde{1}}^{⊤}{\tilde{C}}_{1}\tilde{A}x_{0}\\ {\tilde{L}}_{leg}-{\tilde{1}}^{⊤}{\tilde{C}}_{2}\tilde{A}x_{0} \end{array}\right]$$

(50) $$\begin{array}{cc} H_{1} & =2{\tilde{B}}^{⊤}{\tilde{C}}^{⊤}\tilde{Q}\tilde{C}\tilde{B} \end{array}$$

(51) $$\begin{array}{cc} H_{2} & =2{\tilde{B}}^{⊤}{\tilde{F}}^{⊤}\tilde{T}\tilde{F}\tilde{B} \end{array}$$

(52) $$\begin{array}{cc} H_{3} & =2{\tilde{B}}^{⊤}{\tilde{E}}^{⊤}\tilde{S}\tilde{E}\tilde{B} \end{array}$$

(53) $$\begin{array}{cc} H_{4} & =2{\tilde{B}}^{⊤}{\tilde{G}}^{⊤}\tilde{W}\tilde{G}\tilde{B} \end{array}$$

(54) $$\begin{array}{cc} H_{5} & =2\tilde{R} \end{array}$$

(55) $$\begin{array}{cc} g_{1} & =2{\tilde{B}}^{⊤}{\tilde{C}}^{⊤}\tilde{Q}\left(-P_{CoM,ref}+\tilde{C}\tilde{A}x_{0}+\tilde{D}\right) \end{array}$$

(56) $$\begin{array}{cc} g_{2} & =2{\tilde{B}}^{⊤}{\tilde{F}}^{⊤}\tilde{T}\tilde{F}\tilde{A}x_{0} \end{array}$$

(57) $$\begin{array}{cc} g_{3} & =2{\tilde{B}}^{⊤}{\tilde{E}}^{⊤}\tilde{S}\left(\tilde{E}\tilde{A}x_{0}-{\bar{P}}_{sw,ref}\right) \end{array}$$

(58) $$\begin{array}{cc} g_{4} & =2{\tilde{B}}^{⊤}{\tilde{G}}^{⊤}\tilde{W}\left(\tilde{G}\tilde{A}x_{0}-{\bar{P}}_{b,ref}\right) \end{array}$$

(59) $$\begin{array}{cc} g_{5} & =0 \end{array}$$

## 3. Hierarchical Whole-Body Control

### 3.1. Problem Formulation

The generalized coordinates of the floating base bipedal robot are defined as:

(60) $$q=\left[\begin{array}{c} q_{f}\\ q_{j} \end{array}\right]\in R^{n_{q}}$$

where $q_{f}\in R^{n_{f}}$ represents the position and orientation of the robot’s floating base relative to the inertial coordinate system, and $q_{j}\in R^{n_{j}}$ represents the angle of the actuated joints of the robot and $n_{q}=n_{f}+n_{j}$. The dynamic equation of the system is formulated as

(61) $$M\left(q\right)\ddot{q}+h\left(q,\dot{q}\right)=\left[\begin{array}{c} 0_{n_{f}\times 1}\\ \tau_{j} \end{array}\right]+J_{c}^{⊤}\left(q\right)\omega_{c}$$

where $\dot{q}$ and $\ddot{q}$ are the first and second-order derivative of q, respectively, $M\left(q\right)\in R^{n_{q}\times n_{q}}$ is the mass matrix of the robot, $h\left(q,\dot{q}\right)\in R^{n_{q}}$ collects the Coriolis force, centrifugal force and gravity, and $\tau_{j}\in R^{n_{j}}$ is the joint torque: $\omega_{c,h}={\left[\begin{array}{cc} f_{c,h}^{⊤} & \tau_{c,h}^{⊤} \end{array}\right]}^{⊤}\in R^{6\times 1}$ is the external wrench applied to one leg, including three-dimensional external contact force and three-dimensional contact torque. For the biped robot, $\omega_{c}={\left[\begin{array}{cc} {\omega^{⊤}}_{c,0} & {\omega^{⊤}}_{c,1} \end{array}\right]}^{⊤}\in R^{12\times 1}$. $J_{c}\left(q\right)\in R^{12\times n_{q}}$ is the contact Jacobian matrix.

In order to enable the robot to complete tasks with different priorities, a quadratic programming method with priority hierarchy was proposed [26]. The idea is to put the solution space of the upper priority level as a constraint of the lower level in the optimization problem. The optimization problem of the *i*th level task is defined as:

(62) $$\begin{array}{cc} \min_{\chi_{i}} & {∥\begin{array}{c} {\bar{A}}_{i}\chi_{i}-{\bar{b}}_{i} \end{array}∥}_{Q_{i}}\\ s.t.\;\; & {\bar{\mathrm{ld}}}_{i}⩽{\bar{C}}_{i}\chi_{i}⩽{\bar{\mathrm{ud}}}_{i}\\ \; & {\bar{A}}_{i-1}^{aug}\chi_{i}={\bar{A}}_{i-1}^{aug}\chi_{i-1}^{*}\\ \; & {\bar{\mathrm{ld}}}_{i-1}^{aug}⩽{\bar{C}}_{i-1}^{aug}\chi_{i}⩽{\bar{\mathrm{ud}}}_{i-1}^{aug} \end{array}$$

where ${\bar{A}}_{i}$ and ${\bar{b}}_{i}$ represent the task matrix and target vector corresponding to the current priority. If multiple tasks have the same priority, they could be weighted and combined by a diagonal matrix $Q_{i}$. ${\bar{C}}_{i}$, ${\bar{\mathrm{ld}}}_{i}$ and ${\bar{\mathrm{ud}}}_{i}$ represent the constraint matrix, lower bound, and upper bound of all constraints of the current priority. ${\bar{A}}_{i-1}^{aug}={\left[\begin{array}{ccc} {\bar{A}}_{1}^{⊤} & \cdots & {\bar{A}}_{i-1}^{⊤} \end{array}\right]}^{⊤}$ is the augmented task matrix corresponding to all tasks in the previous priority, and $\chi_{i-1}^{*}$ is the optimal solution of the previous priority. ${\bar{C}}_{i-1}^{aug}$, ${\bar{\mathrm{ld}}}_{i-1}^{aug}$, ${\bar{\mathrm{ud}}}_{i-1}^{aug}$, with a similar form of ${\bar{A}}_{i-1}^{aug}$, represent the augmented constraint matrix and bounds corresponding to all constraints in the previous priority. Here, the tasks in level *i* have higher priorities than tasks in level *j* when i<j. $\chi_{i}$ in level *i* represents the optimization variables to complete the tasks with priorities higher than i+1, which means that the final optimal solution of multi-tasks with priority level $n_{l}$ is $\chi_{n_{l}}^{*}$.

Here, we select the generalized joint acceleration and the contact wrench as the optimization variables in each level:

(63) $$\Upsilon =\left[\begin{array}{c} \ddot{q}\\ \omega_{c} \end{array}\right]$$

### 3.2. Tasks

The definition of each level’s tasks and constraints is given in Table 1. The floating base dynamics task is the basis of all motions of the mobile robot, and all the active forces come from joint motors, so the floating base dynamics task and joint moment constraints are set as the first priority. The linear momentum and trunk posture are critical factors for robot stability, while the ZMP constraint and the plantar friction cone constraint can ensure that the foot of the biped robot will not flip and slip, so these tasks and constraints are set as the second priority. The position of the robot’s feet, posture task, and plantar force screw task are set as the third priority. The detailed definition of each task and constraint is as follows.

#### 3.2.1. Floating Base Dynamics Task

The first six rows of Equation (61) are part of the floating base dynamics, and it can be extracted by a selection matrix $S_{f}$:

(64) $$S_{f}M(q)\ddot{q}+S_{f}h\left(q,\dot{q}\right)=S_{f}\left[\begin{array}{c} 0_{6\times 1}\\ \tau_{j} \end{array}\right]+S_{f}J_{c}^{⊤}\left(q\right)\omega_{c}$$

where $S_{f}=\left[\begin{array}{cc} I_{n_{f}\times n_{f}} & 0_{n_{f}\times n_{j}} \end{array}\right]$. Rearrange Equation (64) into the form of ${\bar{A}}_{i}\chi_{i}={\bar{b}}_{i}$:

(65) $$\left[\begin{array}{cc} S_{f}M & -S_{f}J_{c}^{⊤} \end{array}\right]\left[\begin{array}{c} \ddot{q}\\ \omega_{c} \end{array}\right]=-S_{f}h$$

Therefore, ${\bar{A}}_{0}=\left[\begin{array}{cc} S_{f}M & -S_{f}J^{⊤} \end{array}\right]\in R^{n_{f}\times \left(n_{q}+12\right)}$, ${\bar{b}}_{0}=-S_{f}h\in R^{n_{f}}$.

#### 3.2.2. Centroidal Dynamics Task

Set the desired external force of the centroid task as $f_{G,des}\in R^{3}$, and design the following PD controller to track the centroid task:

(66) $$f_{G,des}=m\left[K_{p,0}\left(p_{G,tar}-p_{G}\right)+K_{d,0}\left({\dot{p}}_{G,tar}-{\dot{p}}_{G}\right)+{\ddot{p}}_{G,tar}\right]$$

(67) $${\bar{A}}_{1,CoM}=\left[\begin{array}{cc} S_{G}A_{G} & 0_{3\times 12} \end{array}\right]\in R^{3\times (n_{q}+12)}$$

(68) $${\bar{b}}_{1,CoM}=f_{G,des}-S_{G}{\dot{A}}_{G}\dot{q}\in R^{3}$$

where $p_{G,tar}\in R^{3}$, ${\dot{p}}_{G,tar}\in R^{3}$ and ${\ddot{p}}_{G,tar}\in R^{3}$ represent the desired position, velocity, and acceleration, respectively; $p_{G}\in R^{3}$, ${\dot{p}}_{G}\in R^{3}$ and ${\ddot{p}}_{G}\in R^{3}$ represent the actual position, velocity, and acceleration, respectively; $S_{G}=\left[\begin{array}{cc} I_{3\times 3} & 0_{3\times 3} \end{array}\right]$ represents the selected matrix, $A_{G}\in R^{6\times n_{q}}$ represents the centroidal momentum matrix [27]; $K_{p,0}\in R^{3\times 3}$ and $K_{d,0}\in R^{3\times 3}$ represent PD gain matrices.

#### 3.2.3. Torso Orientation Task

Set the desired acceleration of the torso orientation task as ${\ddot{\theta }}_{t,des}\in R^{3}$, and design the following PD controller to track the torso orientation:

(69) $${\ddot{\theta }}_{t,des}=K_{p1}\left(\theta_{t,tar}-\theta_{t}\right)+K_{d1}\left({\dot{\theta }}_{t,tar}-{\dot{\theta }}_{t}\right)+{\ddot{\theta }}_{t,tar}$$

where $\theta_{t,tar}\in R^{3}$, ${\dot{\theta }}_{t,tar}\in R^{3}$, and ${\ddot{\theta }}_{t,tar}\in R^{3}$ represent the desired torso orientation angle, angular velocity, and angular acceleration, respectively; $\theta_{t}\in R^{3}$, ${\dot{\theta }}_{t}\in R^{3}$ and ${\ddot{\theta }}_{t}\in R^{3}$ represent the actual torso orientation angle, angular velocity, and angular acceleration, respectively; $K_{p,1}\in R^{3\times 3}$ and $K_{d,1}\in R^{3\times 3}$ represent PD parameter matrices.

The first six rows in the optimization variable Υ represent the position and orientation of the torso, and the fourth to sixth rows are the torso orientation, so, for the torso orientation task:

(70) $${\bar{A}}_{1,torso}=\left[\begin{array}{ccc} 0_{3\times 3} & I_{3\times 3} & 0_{3\times (n_{j}+12)} \end{array}\right]$$

(71) $${\bar{b}}_{1,torso}={\ddot{\theta }}_{t,des}$$

#### 3.2.4. Feet Position and Orientation Task

As above, let ${\ddot{r}}_{f,des}\in R^{12}$ be the desired acceleration of the foot posture, and the PD controller is designed as follows:

(72) $${\ddot{r}}_{f,des}=K_{p2}\left(r_{f,tar}-r_{f}\right)+K_{d2}\left({\dot{r}}_{f,tar}-{\dot{r}}_{f}\right)+{\ddot{r}}_{f,tar}$$

where $r_{f,tar}\in R^{12}$, ${\dot{r}}_{f,tar}\in R^{12}$ and ${\ddot{r}}_{f,tar}\in R^{12}$ represent the desired foot posture, velocity, and acceleration, respectively; $r_{f}\in R^{12}$, ${\dot{r}}_{f}\in R^{12}$ and ${\ddot{r}}_{f}\in R^{12}$ represent the actual foot posture, velocity and acceleration, respectively; $K_{p,2}\in R^{12\times 12}$ and $K_{d,2}\in R^{12\times 12}$ represent PD parameter matrices.

The acceleration of the foot of the end can be obtained according to the following equation:

(73) $$J_{c}\ddot{q}+{\dot{J}}_{c}\dot{q}={\ddot{r}}_{f,des}$$

Then,

(74) $${\bar{A}}_{2,feet}=\left[\begin{array}{cc} J_{c} & 0_{12\times 12} \end{array}\right]\in R^{12\times (n_{q}+12)}$$

(75) $${\bar{b}}_{2,feet}={\ddot{r}}_{f,des}-{\dot{J}}_{c}\dot{q}\in R^{12}$$

#### 3.2.5. Contact Wrench Task

The contact wrench task tracks the desired contact wrench. Let $\omega_{c,des}$ be the desired contact wrench. The last 12 dimensions of the optimization variable Υ are the contact wrench, so for the contact wrench task,

(76) $${\bar{A}}_{2,wrench}=\left[\begin{array}{cc} 0_{12\times n_{q}} & I_{12\times 12} \end{array}\right]$$

(77) $${\bar{b}}_{2,wrench}=\omega_{c,des}\in R^{12}$$

### 3.3. Constraints

#### 3.3.1. Joint Torque Constraint

The last ten rows of Equation (61) represent the actuated joints, which should be limited by torque:

(78) $$S_{j}M\left(q\right)\ddot{q}+S_{j}h\left(q,\dot{q}\right)=S_{j}\left[\begin{array}{c} 0_{6\times 1}\\ \tau_{j} \end{array}\right]+S_{j}J_{c}^{⊤}\left(q\right)\omega_{c}$$

where $S_{j}=\left[\begin{array}{cc} 0_{n_{j}\times n_{f}} & I_{n_{j}\times n_{j}} \end{array}\right]$ represents the selection matrix. The joint torque can be derived as:

(79) $$\tau_{j}=S_{j}M\ddot{q}+S_{j}h-S_{j}J_{c}^{⊤}\omega_{c}$$

Therefore, the joint torque constraint is:

(80) $$-\tau_{limit}\leq \tau_{j}\leq \tau_{limit}$$

where $\tau_{limit}\in R^{n_{j}}$ is the torque limit of each actuated joint. Then, it can be obtained that:

(81) $${\bar{C}}_{0}=\left[\begin{array}{cc} S_{j}M & -S_{j}J_{c}^{⊤} \end{array}\right]\in R^{n_{j}\times \left(n_{q}+12\right)}$$

(82) $${\bar{\mathrm{ld}}}_{0}=-\tau_{limit}-S_{j}h\in R^{n_{j}}$$

(83) $${\bar{\mathrm{ud}}}_{0}=\tau_{limit}-S_{j}h\in R^{n_{j}}$$

#### 3.3.2. ZMP Constraint

According to [28,29], the ZMP constraint on x direction can be written as

(84) $$l_{x}^{-}\leq \frac{-\tau_{c,y}-f_{c,x}d}{f_{c,z}}\leq l_{x}^{+}$$

where $l_{x}^{-}$ and $l_{x}^{+}$ are the minimum and maximum values of ZMP, *d* is the vertical distance between the foot joint and the ground. $\tau_{c,y}$ is the moment applied to the foot in the direction of *y*, $f_{c,x}$, and $f_{c,z}$ represent the force applied to the foot in the direction of *x* and *z*. For the y direction, the ZMP constraint can be derived similarly. Rewrite the ZMP constraints in matrix form, where

(85) $$C_{zmp}=\left[\begin{array}{cccccc} -d & 0 & -l_{x}^{-} & 0 & -1 & 0\\ d & 0 & l_{x}^{+} & 0 & 1 & 0\\ 0 & -d & -l_{y}^{-} & -1 & 0 & 0\\ 0 & d & l_{y}^{+} & 1 & 0 & 0 \end{array}\right]$$

(86) $${\bar{C}}_{1,zmp}=\left[\begin{array}{ccc} 0_{4\times 16} & C_{zmp} & 0_{4\times 6}\\ 0_{4\times 16} & 0_{4\times 6} & C_{zmp} \end{array}\right]$$

(87) $${\bar{\mathrm{ld}}}_{1,zmp}=0_{8\times 1}$$

(88) $${\bar{\mathrm{ud}}}_{1,zmp}=\infty_{8\times 1}$$

#### 3.3.3. Foot Friction Cone Constraint

The stance leg’s ground reaction force should stay in the friction cone to avoid slipping. Here, we employ a pyramid approximation of the friction cone and formulate the foot friction cone constraint as

(89) $$\left|\begin{array}{c} f_{x,y} \end{array}\right|\leq \mu f_{z}$$

where μ is the coefficient of friction; $f_{x,y}$ is the resultant force in the direction of *x* and *y*, and $f_{z}$ is the force in the direction of *z*. Expand and arrange the aforementioned equation into the matrix form:

(90) $$C_{f}=\left[\begin{array}{ccc} 1 & 0 & \mu \\ -1 & 0 & \mu \\ 0 & 1 & \mu \\ 0 & -1 & \mu \\ 0 & 0 & 1 \end{array}\right]$$

(91) $${\bar{C}}_{1,friction}=\left[\begin{array}{ccccc} 0_{5\times 16} & C_{f} & 0_{5\times 3} & 0_{5\times 3} & 0_{5\times 3}\\ 0_{5\times 16} & 0_{5\times 3} & 0_{5\times 3} & C_{f} & 0_{5\times 3} \end{array}\right]$$

(92) $${\bar{\mathrm{lb}}}_{1,friction}=0_{10\times 1}$$

(93) $${\bar{\mathrm{ub}}}_{1,friction}=\infty_{10\times 1}$$

### 3.4. Control Framework

**Figure 2:**
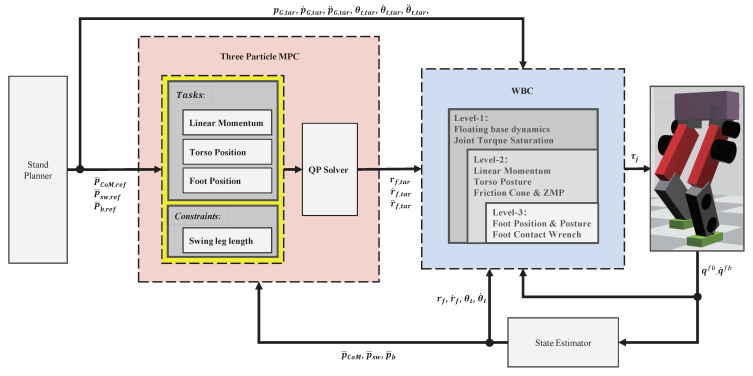
Overall control framework.

Figure 2 shows the overall control framework. Both TP-MPC and WBC run at 1 kHz. The input of TP-MPC is the desired position of CoM, swing leg, and torso in the future *n* steps. WBC then takes the target trajectory of the swing leg calculated by TP-MPC and the target trajectory of CoM and torso orientation given by the stand planner as input. Although TP-MPC can also obtain the trajectories of the torso and CoM, we do not use them in our controller.

## 4. Simulation Results and Discussion

### 4.1. Simulation Setup

The simulation platform is the open-source robot simulator Webots [30]. The control algorithms are implemented with C++. We adopt the rigid body dynamics library (RBDL) [31] to calculate robot kinematics and dynamics and the qpOASES [32] toolkit to solve quadratic programming problems. The simulation environment and the controller run on a computer with an i7-11800H 2.30 GHz processor, 32 GB memory, and RTX3070 8 GB RAM GPU.

The bipedal robot in simulation has ten actuated joints and a total weight of 45 kg. The weights of the torso and a single leg are 22 kg and 11.5 kg, respectively. A single leg accounts for around one-fourth of the robot’s total weight, which implies a significant effect of the swing leg’s motion on the entire robot states. The robot’s soles are 0.22 m-by-0.1 m rectangles.

**Figure 3:**
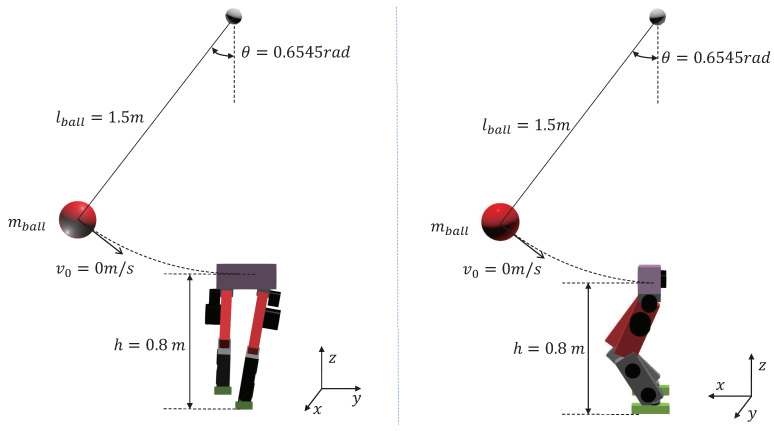
Schematic diagram of collision experiment.

As shown in Figure 3, the biped robot stands on a single leg, and a rigid hanging ball swings down to hit the robot. The suspension point of the ball is vertically above the robot. The ball is released from the same height and angle with zero initial speed. The robot’s torso is hit 0.8 m above the ground in the frontal or the side direction. The initial height of the robot’s CoM is 0.604 m. Therefore, the hit will exert a net torque concerning the robot’s CoM. By increasing the rigid ball’s mass, the impulse received by the robot at the instance of impact increases, meaning larger external disturbance. We compare our control scheme with the WBC-only control scheme in each experiment. For both control schemes, the WBC part is the same. For the WBC-only scheme, the desired torso and leg trajectories remain equal to their initial values.

### 4.2. Results

#### 4.2.1. Frontal Impact

**Figure 4:**
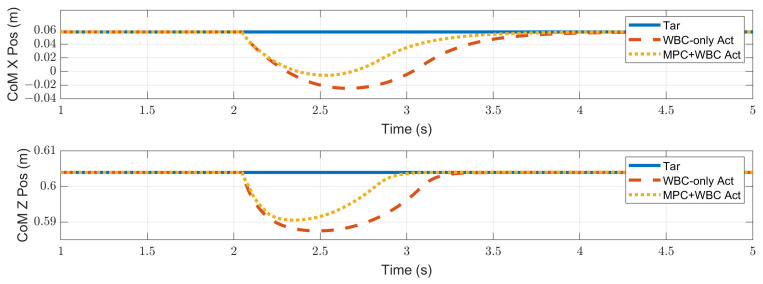
CoM position of the robot under the frontal impact of a 9 kg rigid ball.

**Figure 5:**
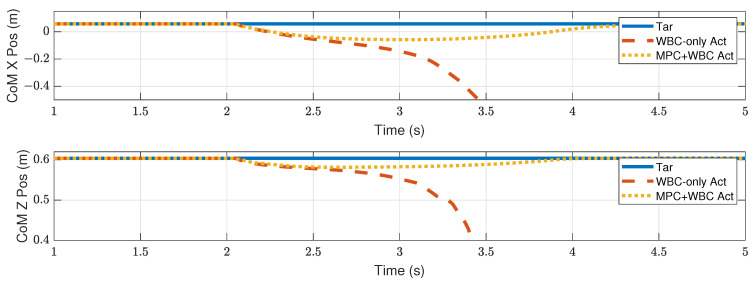
CoM position of the robot under the frontal impact of an 11 kg rigid ball.

**Figure 6:**
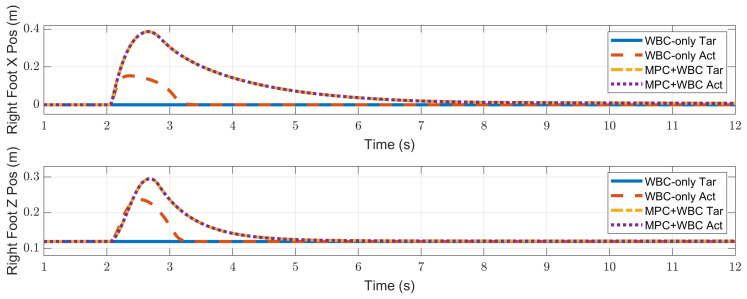
Swing foot position of the robot under the frontal impact of a 9 kg rigid ball.

**Figure 7:**
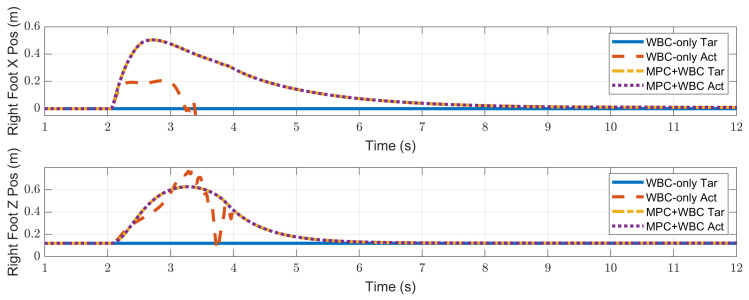
Swing foot position of the robot under the frontal impact of an 11 kg rigid ball.

In the frontal impact experiment, the rigid ball swings along the *x*-axis and hits the center of the torso’s front surface. When the mass of the rigid ball is 9 kg or 11 kg, the robot’s CoM trajectories during the impact and recovery stages are shown in Figure 4 and Figure 5, respectively. In the 9 kg case, both control schemes can resist the external disturbance and bring the robot’s CoM back to its initial position, but our MPC-WBC scheme achieves smaller CoM motions and faster recovery than the WBC-only scheme. For both control schemes, Figure 6 and Figure 7 show the actual trajectories of the swing leg’s foot end and the correspondent reference trajectories given to WBC. For the WBC-only scheme, the WBC’s reference trajectories are constant, while the actual trajectories deviate significantly from the reference. This deviation results from the WBC sacrificing the foot-end tracking task for the high-priority CoM tracking task. For our MPC-WBC scheme, the reference foot end trajectories provided by the MPC and the actual trajectories almost coincide with each other. This suggests that the MPC generates swing foot trajectories that are coherent with the CoM tracking targets. It is also observed that the MPC-WBC scheme generates larger swing foot motions than the WBC-only scheme, which confirms the swing leg’s vital role in balancing the robot.

**Figure 8:**
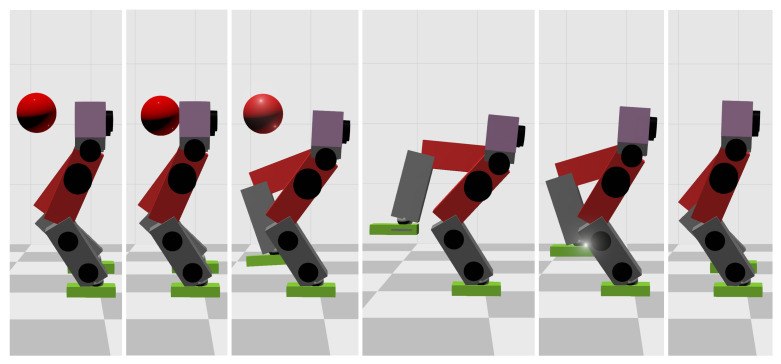
Screenshots of the motions generated by our method in the frontal impact test.

When the ball’s mass increases to 11 kg, the WBC-only scheme causes the divergence of the robot’s CoM trajectories and thus the failure of the entire robot. However, our MPC-WBC scheme can still withstand the impact. Screenshots in Figure 8 depict the robot’s motions during the impact and recovery process under our MPC-WBC scheme. Significant forward and upward motions of the swing leg can be observed after the impact, implying that our method successfully utilizes the swing leg motions to balance the robot.

**Figure 9:**
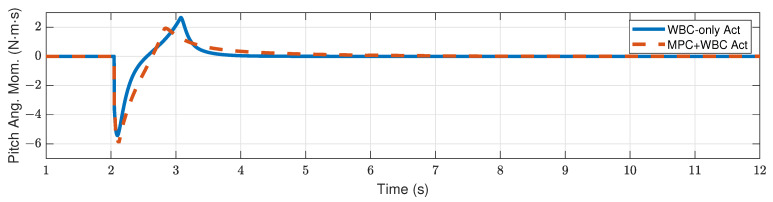
Centroidal pitch angular momentum of the robot under the frontal impact of a 9 kg rigid ball.

**Figure 10:**
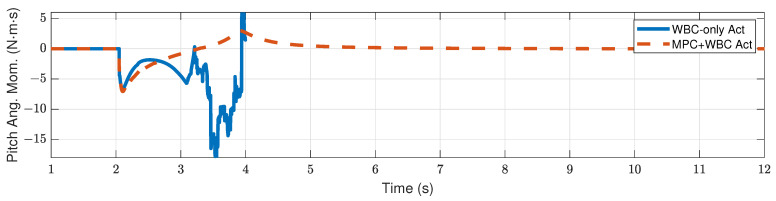
Centroidal pitch angular momentum of the robot under the frontal impact of an 11 kg rigid ball.

Figure 9 and Figure 10 show the robot’s centroidal pitch angular momentum. Although the rotational dynamics are not included in our TP-MPC, the simulation results show that our method is sufficient to deal with a certain degree of torque perturbation relative to the CoM.

**Figure 11:**
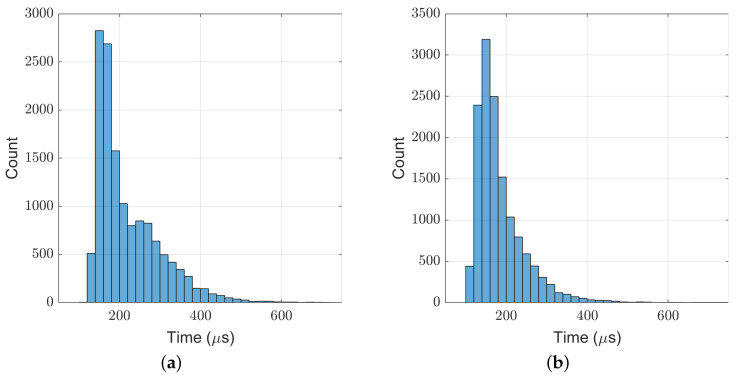
Computation time cost of TP-MPC in the frontal impact test. (**a**) 9 kg ball; (**b**) 11 kg ball.

Figure 11 shows the computation time cost of the TP-MPC algorithm. It can be seen that most of the computations are within 500 μs.

#### 4.2.2. Side Impact

**Figure 12:**
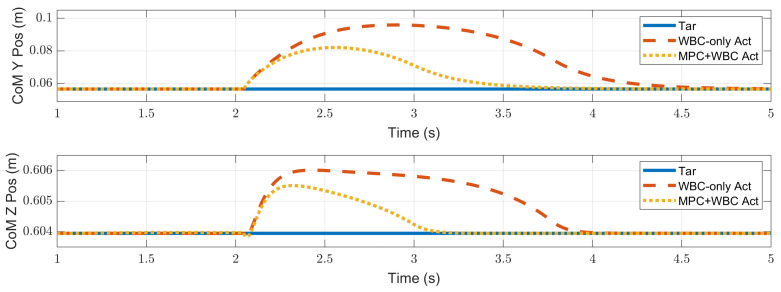
CoM position of the robot under the side impact of a 2.7 kg rigid ball.

**Figure 13:**
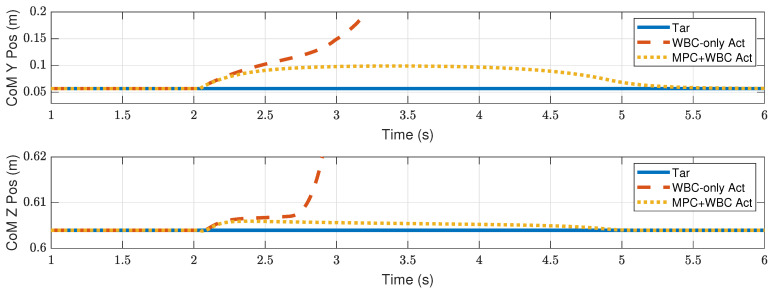
CoM position of the robot under the side impact of a 3.2 kg rigid ball.

**Figure 14:**
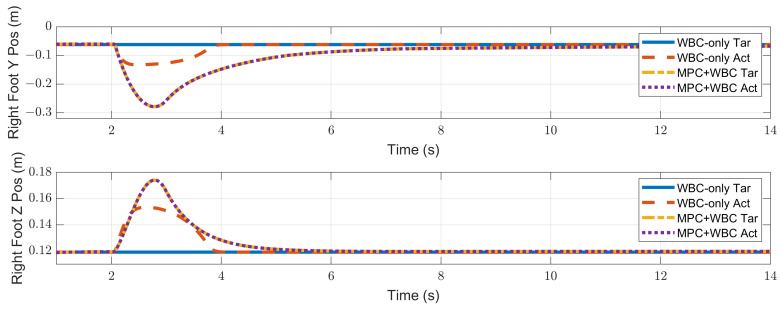
Swing foot position of the robot under the side impact of a 2.7 kg rigid ball.

**Figure 15:**
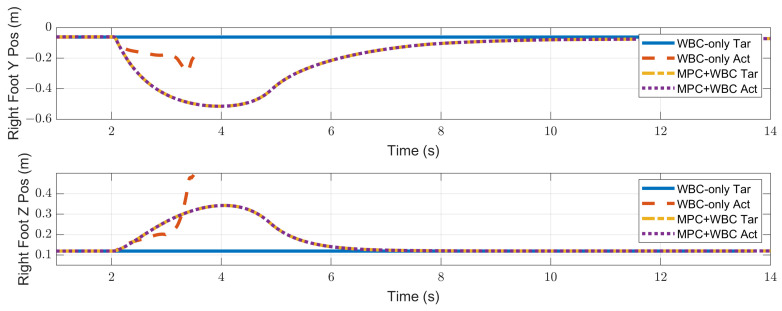
Swing foot position of the robot under the side impact of a 3.2 kg rigid ball.

**Figure 16:**
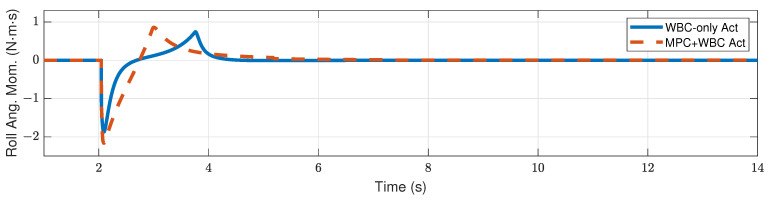
Centroidal roll angular momentum of the robot under the side impact of a 2.7 kg rigid ball.

**Figure 17:**
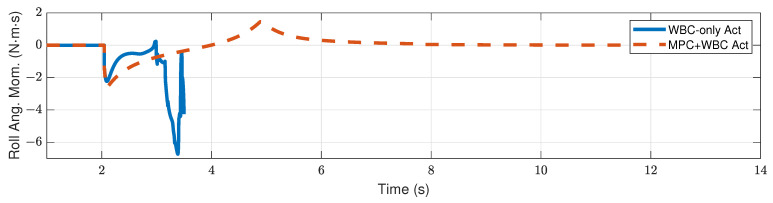
Centroidal roll angular momentum of the robot under the side impact of a 3.2 kg rigid ball.

**Figure 18:**
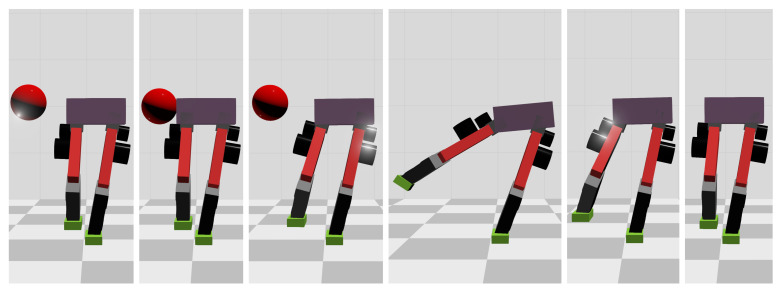
Screenshots of the motions generated by our method in the side impact test.

**Figure 19:**
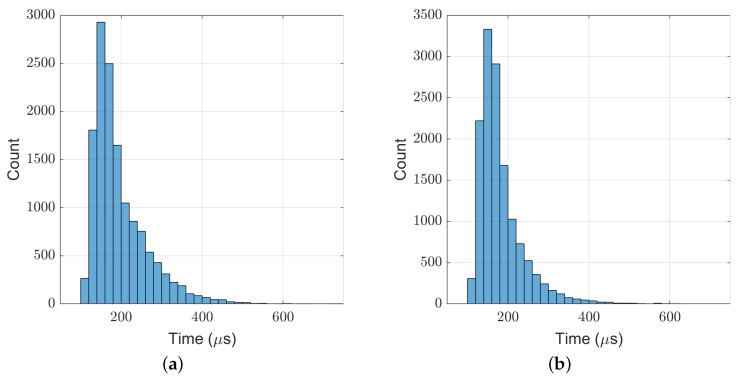
Computation time cost of TP-MPC in the side impact test. (**a**) 2.7 kg ball; (**b**) 3.2 kg ball.

Compared with frontal impact, the robot is more vulnerable to side impact since the supporting polygon is narrower in the side direction. For side impact, when the ball’s mass is 2.7 kg or 3.2 kg, the robot’s CoM trajectories during the impact and recovery stages are shown in Figure 12 and Figure 13, respectively. Figure 14 and Figure 15 show the actual trajectories of the swing leg’s foot end and the correspondent reference trajectories given to WBC. Figure 16 and Figure 17 show the robot’s centroidal roll angular momentum. Similar to the situation of frontal impact in Section 4.2.1, both control schemes can balance the robot under the 2.7 kg ball’s impact, with our MPC-WBC scheme achieving better performance. Under the 3.2 kg ball’s impact, the WBC-only scheme fails, while our scheme still balances the robot. Screenshots in Figure 18 show that the robot can also side-swing its leg under a side impact. Figure 19 shows the computation time cost of the TP-MPC algorithm when the robot is subjected to a side impact.

### 4.3. Discussion

In the WBC, the centroidal dynamics task is considered the second-priority task. Due to its existence, the biped robot can mobilize all joints to maintain the position and velocity of the CoM. Therefore, the WBC-only controller has a certain degree of anti-interference ability. However, it may fail to deal with a considerable impact. The centroidal dynamics task is expressed on the acceleration level. When the acceleration required to achieve the second-priority tasks has been satisfied, the third-priority tasks will be executed as much as possible. That is to say, the WBC will execute the task of swing foot to some extent, even if the robot’s CoM has not returned to its initial state. In the WBC-only control scheme, the desired trajectory of the swing foot only keeps the initial state unchanged, which is almost unhelpful for maintaining the stability of the CoM. On the contrary, in our control scheme, the TP-MPC provides the swing foot with the desired motion trajectory that can balance the position of the CoM. The task of swing foot is almost consistent with the centroidal dynamics task in WBC. Therefore, the control scheme with TP-MPC shows a better ability to maintain the stability of the center of mass.

In the above experiments, the maximum computation time of our TP-MPC is 961 μs, and the average value is about 200 μs. Through proper optimization in coding, our algorithm can run at the frequency of 1 kHz on the real robot.

## 5. Conclusions

This paper proposes the three-particle model predictive control scheme to solve the balance control problem of a bipedal robot standing on one foot. We verified our control scheme in simulation. Compared with the WBC-only control scheme, our scheme can resist much larger disturbances and bring the robot back to its initial state much faster. Although the TP-MPC discards the complicated full dynamics of the robot, it can still catch the main effects of the swing leg motions on the robot’s CoM. Furthermore, since our MPC employs only linear dynamics and constraints, it requires little computational cost, implying real-time execution at the same frequencies as the WBC on a real robot.

Our controller also has some deficiencies. The three-particle model may be over-simplified in some cases, for example, where the robot’s legs or torso have large angular velocities. In the future, we plan to improve our simplified model, for example, including the rigid body’s rotational inertia or adding more particles to the model. We also plan to verify our method on an actual bipedal robot.

## Figures and Tables

**Table 1:** Tasks, Constraints, and Priority Setting of Whole-Body Control for Single Leg Balance Standing.

Priority	Tasks	Tasks Dimension	Constraints	Constraints Dimension
1	Floating Base Dynamics	6	Joint Torque	10
2	Linear Momentum	6	ZMP	9
	Torso Posture		Friction Cone	
3	Foot Position & Posture	16		
	Contact Wrench			

## Data Availability

Not applicable.
